# Health-related quality of life after Gamma Knife radiosurgery in patients with 1–10 brain metastases

**DOI:** 10.1007/s00432-020-03400-w

**Published:** 2020-10-06

**Authors:** Eline Verhaak, Wietske C. M. Schimmel, Karin Gehring, Wilco H. M. Emons, Patrick E. J. Hanssens, Margriet M. Sitskoorn

**Affiliations:** 1 Gamma Knife Center, Elisabeth-TweeSteden Hospital, Tilburg, The Netherlands; 2 Department of Neurosurgery, Elisabeth-TweeSteden Hospital, Tilburg, The Netherlands; 3 Department of Cognitive Neuropsychology, Tilburg University, Warandelaan 2, 5037 AB, Tilburg, The Netherlands; 4 Department of Methodology and Statistics, Tilburg University, Tilburg, The Netherlands

**Keywords:** Brain metastases, Health-related quality of life, Stereotactic radiosurgery, Gamma Knife radiosurgery

## Abstract

**Purpose:**

Increasingly more patients with multiple (> 4) brain metastases (BM) are being treated with stereotactic radiosurgery (SRS). Preserving patients’ health-related quality of life (HRQoL) is an important treatment goal. The aim of this study was to assess (individual) changes in HRQoL in patients with 1–10 BM over time.

**Methods:**

A total of 92 patients were assessed before (*n* = 92) and at 3 (*n* = 66), 6 (*n* = 53), and 9 (*n* = 41) months after Gamma Knife radiosurgery (GKRS), using the Functional Assessment of Cancer Therapy-Brain (FACT-Br). The course of HRQoL was analyzed using linear mixed models. Clinical minimally important differences were used to evaluate individual changes.

**Results:**

At group level, patients’ physical well-being worsened, whereas emotional well-being improved over 9 months. Scores on other HRQoL subscales did not change significantly. Number (1–3 versus 4–10) and volume (small, medium, and large) of BM did not influence HRQoL over time, except for the subscale additional concerns; medium intracranial tumor volume was associated with less additional concerns. On the individual level as well, physical well-being declined while emotional well-being improved in most patients over 9 months after GKRS. At patient level, however, most patients had both declines as well as improvements in the different HRQoL aspects.

**Conclusion:**

Our results indicate that even in patients with up to 10 BM, both at group and individual subscale level, aspects of HRQoL remained stable over nine months after GKRS, except for an improvement in emotional well-being and a decline in physical well-being. Nevertheless, HRQoL scores varied considerably at the individual patient level.

**Trail registration number:**

ClinicalTrials.gov Identifier: NCT02953756, November 3, 2016.

## Introduction

Approximately 10–35% of patients with advanced cancer develop brain metastases (BM) during the course of their disease (Achrol et al. 2019; Arvold et al. 2016). BM are an important cause of morbidity and mortality (Ahluwalia et al. 2014; Tabouret et al. 2012) and the prevalence is rising, mainly due to increased surveillance and improvements in systemic therapies that allow longer survival, which in turn allows for BM to develop (Nayak et al. 2012; Tsao 2015). Preserving health-related quality of life (HRQoL) is a highly important treatment goal in this patient group (Tsao 2015; van der Meer et al. 2018; Wong et al. 2008).

HRQoL is a multidimensional construct that includes, amongst others, health, social, emotional, and functional well-being (Chen et al. 2012). Overall, previous studies demonstrated stable HRQoL in patients with BM after stereotactic radiosurgery (SRS) (Bragstad et al. 2017; Habets et al. 2016; Kirkpatrick et al. 2015; Skeie et al. 2017). However, for the physical aspects of HRQoL, contradictory results have been found (Verhaak et al. 2020). Three studies reported a decline in the physical aspect of HRQoL (Habets et al. 2016; Kotecha et al. 2017; Miller et al. 2017), whereas three other studies reported stable scores over time (Bragstad et al. 2017; Kirkpatrick et al. 2015; Skeie et al. 2017).

SRS is an increasingly used treatment for patients with multiple (> 4) BM. Cumulative intracranial metastatic volume has become a more important criterion than the number of BM when selecting patients eligible for SRS (Hunter et al. 2012; Nabors et al. 2014; Soliman et al. 2016). Thus far, HRQoL has mostly been evaluated in patients with 1–4 BM (in two studies, less than 10% of the patients had 4–6 BM). Number of BM (up to 4) was not predictive of HRQoL over time in previous studies (Bragstad et al. 2017; Habets et al. 2016; Skeie et al. 2017). HRQoL was associated with (change in) Karnofsky Performance Status (KPS): higher KPS was predictive of higher or stable HRQoL (Bragstad et al. 2017; Habets et al. 2016; Skeie et al. 2017; van der Meer et al. 2018), and in two studies, larger baseline volume of BM was associated with worse HRQoL over time (Bragstad et al. 2017; Habets et al. 2016), while in two other studies no such association was found (Skeie et al. 2017; van der Meer et al. 2018).

Group-level changes in HRQoL may mask potential variation over time in HRQoL among individual patients (Verhaak et al. 2020). Van der Meer et al. (2018) showed that HRQoL remained stable at group level 6 months after SRS, but scores varied substantially at the individual level (both at scale and patient level). Despite this study, detailed evaluation of individual HRQoL changes at subscale level remains relatively scarce (Verhaak et al. 2020).

The aim of this study is to examine several aspects of HRQoL at 3, 6, and 9 months after Gamma Knife radiosurgery (GKRS) in patients with up to 10 BM, both at group and individual level. In addition, baseline predictors of change in HRQoL were examined.

## Materials and methods

Data of patients from the prospective longitudinal observational CAR-Study A (ClinicalTrials.gov Identifier: NCT02953756) were analyzed. This study was approved by the Medical Ethics Committee (file NL53472.028.15). Baseline evaluations of cognitive functioning (Schimmel et al. 2019), HRQoL (Verhaak et al. 2019a), and over time evaluations of fatigue (Verhaak et al. 2019b) in our patient group have been previously published.

### Patients and procedures

Adult patients with 1–10 newly diagnosed BM on a contrast-enhanced volumetric MRI scan were recruited. Eligibility criteria were previously published in detail (Verhaak et al. 2019a). Most relevant inclusion criteria were total volume of the BM ≤ 30 cm^3^, KPS ≥ 70, and expected survival > 3 months. Exclusion criteria included small cell lung cancer, meningeal disease, or prior BM treatment.

Assessments (approximately 60 min), including six neuropsychological tests (Wefel et al. 2011) and three self-report questionnaires concerning HRQoL (FACIT.org 2017), fatigue (Smets et al. 1995), and anxiety and depression (Zigmond and Snaith 1983), were performed on the day of and before Gamma Knife radiosurgery (GKRS) and at 3, 6, and 9 months after GKRS. Follow-up assessments were scheduled on the same day and before follow-up MRI scans and consults. All patients gave written informed consent.

### Treatment

Standard SRS procedures were performed with a Leksell Gamma Knife (Elekta AB). All patients received a dose of 18–25 Gy with 99–100% coverage of the target. Dose limits for organs at risk were 18 Gy for the brainstem and 8 Gy for the optic chiasm or optic nerves.

### Measures

Clinical and socio-demographic characteristics were extracted from patients’ medical records.

HRQoL was assessed with the Functional Assessment of Cancer Therapy-Brain (FACT-Br), a self-report questionnaire specific for patients with brain tumors (FACIT.org 2017; Thavarajah et al. 2014; Weitzner et al. 1995). The FACT-Br is commonly used to measure both general HRQoL and specific concerns associated with brain tumors using five subscales. The four (core) subscales of the FACT-Br focus on physical, social, emotional, and functional well-being. The disease-specific subscale ‘additional concerns’ of the FACT-BR was specifically developed for patients with brain tumors. In addition, the following scale scores were calculated: (1) the FACT-General total score, which is based on the first four subscales, measures overall HRQoL and can be used in various groups of patients with cancer; (2) the FACT-Brain total score, which is the FACT-General total score plus the fifth brain tumor-specific subscale (additional concerns); (3) the trial outcome index, which includes the subscales psychical well-being, functional well-being, and additional concerns, is a summary index of physical/functional outcomes (FACIT.org 2017). For all scales, higher scores indicate better HRQoL (Cella et al. 1993; FACIT.org 2017; Thavarajah et al. 2014; Weitzner et al. 1995). Published data from a normative sample, consisting of 1075 persons from the general US adult population (age range = 18–91, 51% female), provided by Brucker et al. (2005), were used to compare HRQoL scores of the patients with BM to.

The total volumetric sum of contrast-enhancing BM was determined at baseline, 3, 6, and 9 months, using T1-weighted MRI scans with 1.5 mm slice thickness. Complete response was defined as a disappearance of all BM (no longer visible). Partial response was defined as a ≥ 65% decrease in total tumor volume and no new BM. Intracranial progression was defined as a ≥ 73% increase in total tumor volume or new BM. Stable disease was defined as no complete response, no partial response, or no intracranial progression (Lin et al. 2015).

### Statistical analyses

Statistical analyses were performed with SPSS version 25 and R (R Core Team), version 3.6.1. To control the false discovery rate due to multiple testing a corrected alpha was used per hypothesis, based on the procedure of Benjamini–Hochberg (Benjamini and Hochberg 1995).

### Group level—status

Descriptive and comparative analyses were performed with respect to the characteristics of patients with at least one follow-up HRQoL assessment and those without. Kaplan–Meier curves and a log rank test were used to analyse differences in overall survival (OS) between patients with and those without at least one follow-up assessment. Group and individual-level changes in HRQoL were determined between baseline and 9 months, and for the three separate time intervals: baseline and 3 months (interval 1), 3 and 6 months (interval 2), and 6 and 9 months (interval 3).

One-sample z tests were used to compare the mean HRQoL scores of the patients at baseline (pre-GRKS) and at 9 months to the mean HRQoL scores of the sample from the general population (Brucker et al. 2005). Glass’ delta effect sizes were calculated (≤ 0.49 small; 0.50–0.79 medium; ≥ 0.80 large effect) (Cohen 1988).

### Group level—change

We used the *nlme* package (Pinheiro et al. 2018) in R (R Core Team) to run a series of linear mixed models (LMMs) of the relationship between each aspect of HRQoL (8 models) and time. To estimate model parameters, the restricted maximum likelihood estimate (REML) method was used. The Akaike Information Criterion (AIC) and Bayesian Information Criterion (BIC) were used to estimate model fit. The intercepts for subjects, of the effect of HRQoL, were added as random intercepts. Random slopes did not improve model fit. The first-order autoregressive covariance structure (AR1) at level 1 and a Scaled Identity matrix at level 2 was used. Additionally, time was included as a categorical variable in subsequent models to examine changes in HRQoL between time intervals.

LMMs were also used to examine interaction effects between time and possible baseline predictors for HRQoL. The following baseline predictors were analyzed: KPS (high ≥ 90 versus low < 90 KPS), systemic treatment before or at time of GKRS (yes versus no), total volume of BM [(small (< 4.8 cm^3^), medium (between 4.8 and 12.6 cm^3^), and large (> 12.6 cm^3^)] (Habets et al. 2016), and number of BM (1–3 versus 4–10 BM).

### Individual level—change

Minimally important differences (MIDs), as provided by Brucker et al. (2005), were used to determine individual clinically meaningful changes in HRQoL. For each time interval, a mean difference of ≥ 2 points for the subscales physical, social, emotional, and functional well-being, and a mean difference of ≥ 5 points for general HRQoL were considered clinically meaningful. For each subscale, numbers of patients with improved, stable, or declined HRQoL were counted at each time interval, except for the FACT-Br total score and trial outcome index. For these scales, no meaningful differences were provided by Brucker et al. (2005).

For the analyses at patient level, four categories were defined based on the MIDs in physical, social, emotional, and functional well-being: (1) “decline” (at least one decline and no improvements on any of these subscales); (2) “improvement” (at least one improvement and no declines); (3) “both” (at least one decline and one improvement); (4) “stable” (no declines and no improvements). Numbers of patients within each category were counted at each time interval.

## Results

### Characteristics, compliance and survival

In total, 92 patients were included. The median overall survival was 11.8 months (95% CI 8.6–15.0 months; 27 patients (29.3%) were alive and censored at time of analysis). Forty percent of patients had more than three BM and the most common histology was non-small cell lung cancer (NSCLC; 60%). Median total volume of BM was 5.64 cm^3^ (Table 1). Follow-up assessments were completed by 66, 53, and 41 patients at 3, 6, and 9 months, respectively. Reasons for non-completion were death (*n* = 24), assessment too burdensome (*n* = 13), no clinical follow-up due to poor neurological or physical condition (*n* = 12), and clinical follow-up in a different hospital (*n* = 2). Of the 66 patients with at least one follow-up, 35 patients (53.0%) had intracranial progression (in 18 patients (51.4%) due to new lesions only), 15 patients (22.7%) had a partial or complete response, and 16 patients (24.2%) had stable disease between time of treatment and last follow-up. There were no statistically significant differences in baseline characteristics between patients with and those without at least one follow-up HRQoL assessment, except for shorter median overall survival (17.1 months, 25 patients (37.9%) were censored, versus 2.7 months, 2 patients (7.7%) were censored, *p* < 0.001). HRQoL scores at baseline were comparable between patients with and those without at least one follow-up HRQoL assessment (Table 1).

**Table 1:** Patient characteristics

	No. of patients included at baseline (%)	No. of patients with ≥ 1 follow-up HRQoL assessment (%)	No. of patients without follow-up HRQoL assessment (baseline only) (%)
Number of participants	92 (100%)	66 (72%)	26 (28%)
Age in years, median (range)	63.0 (31–80)	63.0 (31–80)	61.5 (39–76)
Sex, male	47 (51.1%)	31 (47.0%)	16 (61.5%)
Educational level^a^			
Low	28 (30.4%)	16 (24.2%)	12 (46.2%)
Middle	37 (40.2%)	29 (43.9%)	8 (30.8%)
High	27 (29.3%)	21 (31.8%)	6 (23.1%)
KPS, median (range)	90 (70–100)	90 (70–100)	90 (70–100)
70–80	33 (35.9%)	21 (31.8%)	12 (46.2%)
90–100	59 (64.1%)	45 (68.2%)	14 (53.8%)
RPA			
Class 1	16 (17.4%)	13 (19.7%)	3 (11.5%)
Class 2	76 (82.6%)	53 (80.3%)	23 (88.5%)
GPA			
Class 2	15 (16.3%)	12 (18.2%)	3 (11.5%)
Class 3	60 (65.2%)	42 (63.6%)	18 (69.2%)
Class 4	17 (18.5%)	12 (18.2%)	5 (19.2%)
Number of BM			
1–3	55 (59.8%)	41 (62.1%)	14 (53.8%)
4–10	37 (40.2%)	25 (37.9%)	12 (46.2%)
Total volume of BM cm^3^, median (range)^b^	5.6 (0.02–31.1)	5.4 (0.02–31.1)	5.8 (0.04–31.0)
Small (< 4.8 cm^3^)	40 (43.5%)	29 (43.9%)	11 (42.3%)
Middle (4.8–12.6 cm^3^)	25 (27.2%)	17 (25.8%)	8 (30.8%)
Large (> 12.6 cm^3^)	27 (29.3%)	20 (30.3%)	7 (26.9%)
Primary site			
Lung (NSCLC)	55 (59.8%)	40 (60.6%)	15 (57.7%)
Renal	15 (16.3%)	12 (18.2%)	3 (11.5%)
Melanoma	12 (13.0%)	6 (9.1%)	6 (23.1%)
Breast	6 (6.5%)	5 (7.6%)	1 (3.8%)
Other	4 (4.3%)	3 (4.5%)	1 (3.8%)
Systemic therapy^c^			
No	39 (42.4%)	27 (40.9%)	12 (46.2%)
Yes	53 (57.6%)	39 (59.1%)	14 (53.8%)
Overall survival in months, median (range)	11.8 (8.6 to 15.0)	17.1 (10.5 to 23.7)	2.7 (1.7 to 3.7)
HRQoL			
Physical well-being, mean (SD)	22.7 (4.8)	22.8 (4.9)	22.3 (4.6)
Social well-being, mean (SD)	23.0 (5.3)	22.9 (5.1)	23.2 (5.9)
Emotional well-being, mean (SD)	16.0 (4.7)	16.1 (4.4)	15.8 (5.5)
Functional well-being, mean (SD)	17.9 (6.1)	18.5 (5.3)	16.3 (7.6)
FACT-General, mean (SD)	79.6 (15.6)	80.4 (13.9)	77.7 (19.5)
Additional concerns, mean (SD)	50.5 (11.2)	51.1 (11.5)	49.0 (10.5)
FACT-Brain, mean (SD)	130.1 (24.0)	131.5 (22.2)	126.7 (28.3)
Trial outcome index, mean (SD)	91.1 (18.8)	92.5 (18.1)	87.6 (20.4)

*BM* brain metastases, *FACT-Brain* Functional Assessment of Cancer Therapy-Brain, *FACT-General* Functional Assessment of Cancer Therapy-General, *GPA* graded prognostic assessment, *HRQoL* health-related quality of life, *KPS* Karnofsky Performance Status, *No.* number, *NSCLC* non-small cell lung cancer, *RPA* recursive partitioning analysis, *SD* standard deviation

^a^Educational level according to Verhage (Verhage 1964) (7 levels): low = 1–4, middle = 5, high = 6–7

^b^Total volume of brain metastases by patient (two patients had a total volume of BM > 30 cm^3^, 31.1 and 31.0 cm^3^, on the MRI-scan used for treatment planning)

^c^Before or at time of Gamma Knife radiosurgery

### Health-related quality of life at baseline and at 9 months (status)

Both at baseline and at 9 months after GKRS, patients with BM had on average better social well-being and worse emotional well-being compared to the general American population (Brucker et al. 2005). There were no significant differences for physical well-being, functional well-being, and FACT-General (Table 2).

**Table 2:** Health-related quality of life of patients with BM

HRQoL subscales	General population (*n* = 1075)^a^ Mean (SD)	Patients with BM Mean raw HRQoL scores (SD)				Patients with BM versus general population^a^					
						Baseline			9 months after GKRS		
		Baseline (n = 92)	3 months (*n* = 66)	6 months (*n* = 53)	9 months (*n* = 41)	z value	p*	Effect size	z value	p*	Effect size
Physical well-being	22.7 (5.4)	22.7 (4.8)	20.7 (5.6)	21.0 (5.1)	21.2 (5.1)	0.00	1.000	.00	− 1.78	.075	.28
Social well-being	19.1 (6.8)	23.0 (5.3)	23.1 (4.0)	22.6 (5.0)	23.1 (3.4)	5.50	** < .001**	.57	3.77	** < .001**	.59
Emotional well-being	19.9 (4.8)	16.0 (4.7)	17.5 (3.8)	18.2 (4.5)	18.0 (4.3)	− 7.79	** < .001**	.81	− 2.53	**.011**	.40
Functional well-being	18.5 (6.8)	17.9 (6.1)	17.3 (5.5)	17.6 (5.8)	18.7 (5.7)	− 0.85	.397	.09	0.19	.851	.03
FACT-General	80.1 (18.1)	79.6 (15.6)	78.5 (14.2)	79.3 (14.3)	81.0 (14.3)	− 0.26	.791	.03	0.32	.750	.05
Additional concerns^b^		50.5 (11.2)	52.2 (11.3)	52.8 (10.6)	53.7 (10.4)						
FACT-Brain^b^		130.1 (24.0)	130.7 (22.9)	132.1 (22.6)	134.7 (21.7)						
Trial outcome index^b^		91.1 (18.8)	90.1 (19.2)	91.3 (18.2)	93.6 (17.3)						

*BM* brain metastases, *FACT-Brain* Functional Assessment of Cancer Therapy-Brain, *FACT-General* Functional Assessment of Cancer Therapy-General, *GKRS* Gamma Knife radiosurgery, *HRQoL* health-related quality of life, *n* number of patients,* SD* standard deviation

^a^Normative data of general population (*n* = 1075) of Brucker et al. (2005), ^b^Normative data not available/applicable. *Corrected alpha of 0.02 using the Benjamini–Hochberg procedure (Benjamini and Hochberg 1995). Bold text indicates statistical significance. Higher scores indicate better HRQoL (Cella et al. 1993; FACIT.org 2017; Thavarajah et al. 2014; Weitzner et al. 1995)

### Health-related quality of life over time—group level

Over the course of 9 months, patients’ physical well-being worsened significantly whereas patients’ emotional well-being improved significantly. More specifically, between baseline and 3 months, there was a significant decrease in physical well-being and an increase in emotional well-being, after which scores did not change significantly between 3 and 6 months, nor between 6 and 9 months (Table 3). No significant change between baseline and the 9-month assessment was found for all other scales of the FACT-Br (Table 3). Analyses of interactions of time with baseline predictors demonstrated that patients with low (versus high) KPS had a significantly larger improvement in emotional well-being over time. Patients with medium intracranial tumor volumes at baseline had significantly less additional concerns over time compared to patients with small or large intracranial tumor volumes (Table 3).

**Table 3:** Course and predictors of the HRQoL of patients with BM over time

		Physical well-being	Social well-being	Emotional well-being	Functional well-being	FACT-General	Additional concerns	FACT-Brain	Trial outcome index
*Time slope*									
Time slope T0–T9	*b* (SE)	− 0.68 (0.3)	0.02 (0.2)	0.74 (0.2)	− 0.05 (0.3)	0.03 (0.6)	0.75 (0.4)	0.71 (0.9)	− 0.07 (0.7)
	*F* value	6.394	0.006	17.138	0.044	0.002	3.482	0.691	0.010
	*p* value*	**.012**	.939	** < .001**	.834	.966	.064	.407	.921
Interval T0–T3	*b *(SE)	**− 2.12 (0.6)**	0.10 (0.6)	**1.46 (0.5)**	− 1.01 (0.6)	− 1.63 (1.5)	1.23 (0.9)	− 0.46 (2.0)	− 2.01 (1.7)
Interval T3–T6	*b *(SE)	0.46 (0.7)	− 0.27 (0.6)	0.90 (0.5)	0.60 (0.7)	1.89 (1.6)	1.12 (1.0)	3.04 (2.2)	2.26 (1.8)
Interval T6–T9	*b *(SE)	− 0.28 (0.8)	0.34 (0.7)	− 0.49 (0.6)	0.27 (0.8)	− 0.48 (1.9)	− 0.45 (1.2)	− 1.05 (2.5)	− 0.73 (2.1)
*Interaction effect with time*									
KPS 70–80 vs. 90–100 (ref)	*b*	0.304	− 0.410	1.166	0.439	1.193	1.399	2.430	1.979
	SE	0.63	0.53	0.42	0.63	1.47	0.94	2.10	1.70
	*F *value	0.232	0.600	7.639	0.490	0.660	2.198	1.343	1.349
	*p* value*	.631	.440	**.006**	.485	.418	.140	.248	.247
Systemic treatment Yes vs. no (ref)	*b*	0.759	0.963	− 0.575	0.165	1.464	0.406	1.955	1.377
	SE	0.59	0.49	0.39	0.58	1.36	0.87	1.94	1.58
	*F* value	1.664	3.868	2.189	0.081	1.160	0.216	1.011	0.759
	*p* value*	.199	.051	.141	.776	.283	.643	.316	.385
Large intracranial tumor volume Large vs. medium (ref)	*b*	− 1.622	0.142	0.232	− 0.489	− 1.815	− 2.684	− 4.581	− 4.962
	SE	0.73	0.61	0.48	0.72	1.68	1.08	2.39	1.94
	*F *value	4.984	0.055	0.234	0.466	1.174	6.223	3.665	6.509
	*p* value*	.027	.815	.629	.496	.280	**.014**	.057	.012
Small intracranial tumor volume Small vs medium (ref)	*b*	− 0.648	0.496	0.134	− 0.151	− 0.124	− 2.508	− 2.633	− 3.273
	SE	0.67	0.56	0.44	0.66	1.53	0.99	2.19	1.78
	*F* value	0.933	0.797	0.094	0.053	0.007	6.483	1.442	3.371
	*p* value*	.336	.373	.759	.819	.936	**.012**	.232	.068
Number of BM 1–3 (ref) versus 4–10	*b*	− 0.349	0.284	0.659	− 0.543	0.206	− 1.580	− 1.262	− 2.243
	SE	0.56	0.47	0.37	0.55	1.29	0.83	1.84	1.50
	*F* value	0.386	0.371	3.206	0.970	0.025	3.641	0.469	2.241
	*p* value*	.536	.544	.075	.326	.874	.058	.495	.136

*BM* brain metastases, *CI* confidence interval, *HRQoL* health-related quality of life, *KPS* Karnofsky Performance Status, *ref* reference category, *SE* standard error, *T0* baseline, *T3* 3 months, *T6* 6 months, *T9* 9 months, *vs* versus

*Corrected alphas, using the Benjamini–Hochberg procedure (Benjamini and Hochberg 1995), were 0.013 for the overall models (time slope T0–T9), .017 for the time intervals of each HRQoL subscale, and .020 for the predictors of additional concerns and .010 for the predictors of the other HRQoL scales. Bold text indicates statistical significance

### Health-related quality of life over time—individual level

**Fig. 1:**
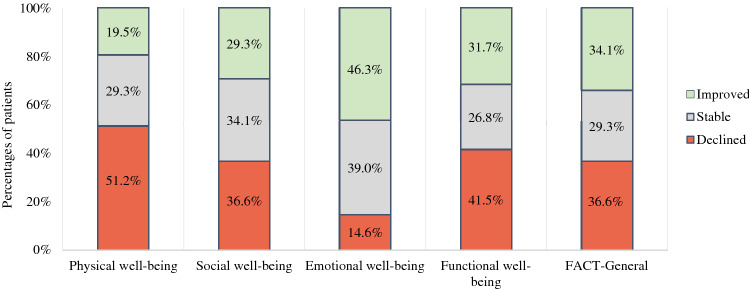
Clinically meaningful changes in HRQoL in patients with BM from baseline to 9 months after GKRS at subscale level. *BM* brain metastases, *FACT-G* Functional Assessment of Cancer Therapy-General, *GKRS* Gamma Knife radiosurgery, *HRQoL* health-related quality of life, *WB* well-being. The number of patients = 41

At the subscale level, for most patients (58.5–85.3%), scores remained stable or improved over 9 months after GKRS, except for physical well-being: scores on this subscale declined in 51.2% of patients. Decline in physical well-being was most pronounced between baseline and 3 months (45.5%) and between 6 and 9 months (41.5%) (Table 4). Between 6 and 9 months, a comparable number of patients showed an improvement (36.6%) in physical well-being. Regarding emotional well-being, scores improved in 46.3% of the patients and remained stable in 39.0% of the patients (Fig. 1). Improvement occurred especially during the first two intervals (T0–T3: 50.0% and T3–T6: 43.4%) (Table 4). Still, substantial groups of patients showed declines in social (36.6%) and functional (41.5%) well-being, and overall HRQoL (36.6%). Proportions of patients with declined, improved, and stable scores were comparable for these scales (Fig. 1).

**Table 4:** Clinically meaningful changes in HRQoL in patients with BM after GKRS at the subscale level

HRQoL subscales	T0–T9 *n* = 41			T0–T3 *n* = 66			T3–T6 *N* = 53			T6–T9 *n* = 41		
	Declined *n* (%)	Stable *n* (%)	Improved *n* (%)	Declined *n* (%)	Stable *n* (%)	Improved *n* (%)	Declined *n* (%)	Stable *n* (%)	Improved *n* (%)	Declined *n* (%)	Stable *n* (%)	Improved *n* (%)
Physical well-being	21 (51.2)	12 (29.3)	8 (19.5)	30 (45.5)	27 (40.9)	9 (13.6)	15 (28.3)	25 (47.2)	13 (24.5)	17 (41.5)	9 (22.0)	15 (36.6)
Social well-being	15 (36.6)	14 (34.1)	12 (29.3)	22 (33.3)	28 (42.4)	16 (24.2)	15 (28.3)	21 (39.6)	17 (32.1)	11 (26.8)	20 (48.8)	10 (24.4)
Emotional well-being	6 (14.6)	16 (39.0)	19 (46.3)	13 (19.7)	20 (30.3)	33 (50.0)	10 (18.9)	20 (37.7)	23 (43.4)	13 (31.7)	21 (51.2)	7 (17.1)
Functional well-being	17 (41.5)	11 (26.8)	13 (31.7)	30 (45.5)	17 (25.8)	19 (28.8)	14 (26.4)	24 (45.3)	15 (28.3)	14 (34.1)	12 (29.3)	15 (36.6)
FACT-General	15 (36.6)	12 (29.3)	14 (34.1)	24 (36.4)	23 (34.8)	19 (28.8)	14 (26.4)	21 (39.6)	18 (34.0)	14 (34.1)	13 (31.7)	14 (34.1)

Differences of ≥ 2 points in physical, social, emotional, and functional well-being and differences of ≥ 5 points in general HRQoL were considered clinically meaningful, based on normative data of Brucker et al. (2005)

*BM* brain metastases, *FACT-General* Functional Assessment of Cancer Therapy-General, *GKRS* Gamma Knife radiosurgery, *HRQoL* health-related quality of life, *T0* baseline, *T3* 3 months, *T6* 6 months, *T9* 9 months

**Fig. 2:**
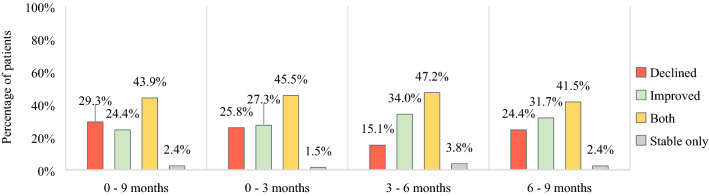
Clinically meaningful changes in HRQoL in patients with BM after GKRS at patient level. *BM* brain metastases, *GKRS* Gamma Knife radiosurgery, *HRQoL* health-related quality of life, *n* number of patients. *n* = 41 for 0–9 months and 6–9 months, *n* = 66 for 0–3 months, *n* = 53 for 3–6 months

At the patient level, over 9 months as well as between the intermediate assessments, most patients (41.5–47.2%) had both declines and improvements in different aspects of HRQoL. Smaller proportions of patients had only one or more declines in HRQoL aspects (15.1–29.3%), or only one or more improvements (24.4–34.0%). Almost none of the patients (1.5–3.8% between all intermediate measurements) showed no clinically meaningful changes at all on any of the HRQoL aspects (Fig. 2).

## Discussion

The aim of this study was to evaluate the different aspects of HRQoL up to 9 months after GKRS in patients with 1–10 BM, both at group and individual level. Before, and at nine months after GKRS, compared to general population norms, patients’ emotional well-being was significantly lower whereas their social well-being was significantly higher. Worse emotional well-being before SRS (compared to the general population) has also been reported in a previous study in a group of patients with 1–4 BM (Habets et al. 2016). Emotional distress could be caused by the recent diagnosis of a serious life-threatening disease and the upcoming treatment (Verhaak et al. 2019a). On the other hand, patients may have experienced better social well-being due to strong social support from family, friends, and other groups, just before the upcoming treatment (Verhaak et al. 2019a) and at follow-up (Applebaum et al. 2014).

At group level, patients’ HRQoL remained stable over nine months, except for a significant decline in physical well-being and a significant improvement in emotional well-being, which both occurred during the first 3 months after GKRS. Previous studies also found stable HRQoL over time (Bragstad et al. 2017; Habets et al. 2016; Kirkpatrick et al. 2015; Skeie et al. 2017). In three of these studies, however, no decline in physical well-being was found (Bragstad et al. 2017; Kirkpatrick et al. 2015; Skeie et al. 2017), except for a trend towards a decline in physical well-being in one study (*n* = 24) (Kirkpatrick et al. 2015). Differences between studies may be explained by the inclusion of patients with a different initial performance status [KPS ≥ 70 (Kirkpatrick et al. 2015) versus KPS ≥ 60 (Bragstad et al. 2017; Skeie et al. 2017)]. Studies including patients with a lower initial KPS [KPS ≥ 60 (Bragstad et al. 2017; Skeie et al. 2017)] reported lower mean baseline physical well-being compared to studies including patients with a higher initial KPS [KPS ≥ 70 (Kirkpatrick et al. 2015)], as in our study. Mean physical well-being at 3, 6, and 9 months appeared to be comparable between studies.

In line with our group-level results, and the individual results in the study of van der Meer et al. (2018), for most individual patients HRQoL aspects remained stable or improved over 9 months after GKRS, except for a decline in physical well-being. This decline was most prominent during the first 3 months, and from 6 to 9 months after GKRS. Additionally, in line with our group results, improvement in emotional well-being was most pronounced in the early phase after GKRS.

There was, however, a degree of individual variation in HRQoL, both at subscale and patient level, that was not reflected in the group-level results. This was most clearly visible at patient level: most patients had both declines as well as improvements in the different aspects of HRQoL. These individual HRQoL changes within patients were masked at group level, which underlines the relevance of analyzing data both at group and at the individual patient level. Furthermore, our results showed that although patients’ general HRQoL (i.e., the FACT-G that is comprised of the four main HRQoL subscales) remained stable at group level over 9 months, scores on physical and emotional well-being changed considerably during this period (at group and individual subscale level). This supports the notion that HRQoL is a complex construct, and to apprehend the multifaceted nature of it, the different aspects of HRQoL should be measured and analyzed separately. Combining (subscale) sores into a total score (e.g., the FACT-G) may potentially mask particular issues concerning the different aspects of HRQoL (Verhaak et al. 2020).

The selected pre-GKRS predictors influenced scores on two out of eight aspects of HRQoL; lower baseline KPS was associated with significantly more improvement in emotional well-being over time, and medium baseline intracranial tumor volume was associated with less additional concerns over time. In line with previous studies, baseline number of BM did not influence HRQoL (Bragstad et al. 2017; Habets et al. 2016; Skeie et al. 2017) over time.

As mentioned before, maintaining HRQoL after treatment is a very important treatment goal. Our results indicate that HRQoL levels after GKRS are largely maintained except for a decline in physical well-being. This aspect includes questions regarding a lack of energy, pain, being bothered by side effects, feeling ill, and trouble meeting the needs of their family due to the physical condition. Especially in the first phase after treatment, patients reported a decline in physical well-being. In this early phase, these patients also experienced more physical fatigue as was reported in our previous publication (Verhaak et al. 2019b). This decline in physical well-being can have a large negative impact on a patient’s daily life; fatigue, pain and being bothered by side effects can cause patients to withdraw themselves from social activities, and not being able to physically provide for the needs of family and friends can have a negative effect on self-esteem. For patients experiencing problems with (declined) physical well-being, interventions such as psychoeducation, energy conservation, and coping strategies (Ahlberg et al. 2003; Day et al. 2016) might be helpful.

This study has several limitations. A heterogeneous study sample of patients with BM originating from several types of primary cancers was included. Specific systemic treatments, symptoms, and side effects related to the different primary cancers might have influenced HRQoL differently. Additionally, self-reported HRQoL may be positively or negatively biased by how a patient is feeling at the time of questionnaire administration (Demetriou et al. 2015). These feelings might not only be related to treatment factors but also to personal factors. Lastly, although a standard deviation of 0.5 is commonly used as cutoff for a minimally important difference, it is also considered a conservative estimate of a minimally important difference (Brucker et al. 2005; Ferrer and Pardo 2014; Norman et al. 2003). We used cut-offs provided by Brucker et al. (2005), based on clinical and subjective indicators, to determine individual meaningful differences specifically for the FACT-G. Using these cut-offs, which are close to 0.3 standard deviation, we may, however, have overestimated individual meaningful change in our sample. Additionally, there were no normative values available for the subscale additional concerns (and consequently for the FACT-Br total score and trial outcome index) based on the general population (Brucker et al. 2005), as this scale includes questions that are relevant for patients with brain tumors only.

Future studies are needed to evaluate the effect of psychological factors such as fatigue, mood, coping style, and personality, on HRQoL over time, as treatment and disease-related factors did not have substantial predictive value in this study. To better understand the influence of the different primary cancers on HRQoL, patients with BM should be analyzed separately based on their primary tumors.

## Conclusion

Our results show that, even in patients with up to 10 BM, the number of BM did not influence change in HRQoL after GKRS, which is in line with previous studies in patients with mostly up to 4 BM. Both at group and at the individual subscale level, aspects of HRQoL remained stable or improved over 9 months after GKRS, except for a decline in physical well-being. There was, however, a degree of individual variation in HRQoL at patient level that was masked at group level. In terms of HRQoL, GKRS should be considered a treatment option for patients with up to 10 BM.

## Data Availability

The datasets generated during and/or analyzed during the current study are available from the corresponding author on reasonable request.
